# Correlative Raman Spectroscopy–SEM Investigations of Sintered Magnesium–Calcium Alloys for Biomedical Applications

**DOI:** 10.3390/ma18163873

**Published:** 2025-08-18

**Authors:** Eshwara Nidadavolu, Martin Mikulics, Martin Wolff, Thomas Ebel, Regine Willumeit-Römer, Berit Zeller-Plumhoff, Joachim Mayer, Hilde Helen Hardtdegen

**Affiliations:** 1 Helmholtz-Zentrum Hereon GmbH, Max-Planck Straße 1, 21502 Geesthacht, Germany; martin.wolff@hereon.de (M.W.); thomas.ebel0512@googlemail.com (T.E.); regine.willumeit@hereon.de (R.W.-R.); berit.zeller-plumhoff@hereon.de (B.Z.-P.); 2 Ernst-Ruska-Centre (ER-C-2), Forschungszentrum Jülich, 52425 Jülich, Germany; m.mikulics@fz-juelich.de (M.M.); j.mayer@fz-juelich.de (J.M.); h.hardtdegen@fz-juelich.de (H.H.H.); 3 Institute of Materials Science, Faculty of Engineering, Christian Albrecht University of Kiel (CAU), 24118 Kiel, Germany; 4 Chair for Data-Driven Analysis and Design of Materials, Faculty of Mechanical Engineering and Marine Technologies, University of Rostock, 18051 Rostock, Germany; 5 Central Facility for Electron Microscopy (GFE), RWTH Aachen University, 52074 Aachen, Germany

**Keywords:** Mg-Ca alloys, correlative characterization, metal injection molding, sintering, Raman spectroscopy, scanning electron microscopy, microstructure

## Abstract

In this study, a correlative approach using Raman spectroscopy and scanning electron microscopy (SEM) is introduced to meet the challenges of identifying impurities, especially carbon-related compounds in metal injection-molded (MIM) Mg-0.6Ca specimens designed for biomedical applications. This study addresses, for the first time, the issue of carbon residuals in the binder-based powder metallurgy (PM) processing of Mg-0.6Ca materials. A deeper understanding of the material microstructure is important to assess the microstructure homogeneity at submicron levels as this later affects material degradation and biocompatibility behavior. Both spectroscopic and microscopic techniques used in this study respond to the concerns of secondary phase distributions and their possible stoichiometry. Our micro-Raman measurements performed over a large area reveal Raman modes at ~1370 cm^−1^ and ~1560 cm^−1^, which are ascribed to the elemental carbon, and at ~1865 cm^−1^, related to C≡C stretching modes. Our study found that these carbonaceous residuals/contaminations in the material microstructure originated from the polymeric binder components used in the MIM fabrication route, which then react with the base material components, including impurities, at elevated thermal debinding and sintering temperatures. Additionally, using evidence from the literature on thermal carbon cracking, the presence of both free carbon and calcium carbide phases is inferred in the sintered Mg-0.6Ca material in addition to the Mg_2_Ca, oxide, and silicate phases. This first-of-its-kind correlative characterization approach for PM-processed Mg biomaterials is fast, non-destructive, and provides deeper knowledge on the formed residual carbonaceous phases. This is crucial in Mg alloy development strategies to ensure reproducible in vitro degradation and cell adhesion characteristics for the next generation of biocompatible magnesium materials.

## 1. Introduction

A large amount of work was carried out over the past decade on the development of biodegradable magnesium (Mg)-based materials, and this interest is only growing [1,2,3]. Due to their biocompatibility, physiological degradation characteristics, and mechanical property profiles like high strength-to-weight ratio, they are potential candidates for biomedical implant applications [4,5,6,7]. A vast amount of the literature indicates that the choice of alloying elements, processing conditions, coatings, and impurity concentrations affects the final material properties that are crucial in avoiding uncontrollable and/or unwanted electrochemical reactions during Mg degradation under physiological conditions. Importantly, microstructural entities, like grain size, porosity, secondary phases, and impurity particles, contribute to both degradation and cell adhesion characteristics [8,9,10,11]. Therefore, various processing techniques have evolved with time to produce a homogenous microstructure. Amongst them, binder-based PM techniques, like MIM, 3D printing, etc., are promising in producing near-net shape implant materials based on Mg. Additionally, the binder-based 3D printing of Mg materials facilitates the production of patient-specific Mg implant designs [12,13,14].

At times, however, the unintentional formation of compounds or phases due to the added constituents during raw material production or material fabrication may hinder achieving optimal property profiles for the biomedical functioning of Mg materials. This requires occasional revisiting of the process chain for continuous improvements to achieve reproducible results. A good place to start is the material microstructure because most of the process variations, like the choice of the initial powder sizes, impurities, storage conditions, sintering or heat treatment conditions, and binder compositions, are reflected in the final microstructure.

In the present study, MIM-processed Mg-0.6Ca material is selected for its good sintering characteristics, which are reflected in the specimen’s high relative sinter densities and homogeneous microstructures [15,16,17]. In addition, Ca micro-alloying (<1 wt.%) is reported to improve degradation resistance in Mg while maintaining adequate mechanical properties [18,19,20]. The MIM technique involves the use of a binder system (composed of polymers and paraffin waxes) that is mixed with raw Mg alloy powders to facilitate the flow characteristics necessary for injection. Even though the subsequent solvent debinding and thermal debinding treatments prior to sintering should remove these polymeric-based binder materials, they might still represent a source for carbon contamination by incorporating themselves as phases into the final product during sintering [21,22]. They could potentially be, by nature, non-biocompatible and/or act as catalysts for uncontrolled physiological degradation through the formation of various carbon-related phases with Mg, Ca, or other impurities in the material during processing [23,24,25]. The literature reports good cell biocompatibility for Mg materials processed via the extrusion technique [26,27]. Additionally, in our internal works, good cell viability is observed on the sintered surfaces of the pressed and sintered Mg materials (without polymeric binder involvement), unlike Mg materials that are processed using binder components, for example, in MIM and 3D-printing techniques. Mg materials processed via binder-based techniques often showed non-reproducible cell viability behavior. This indicates a certain toxicity in the sintered Mg materials produced via MIM or 3D-printing techniques that contain the polymeric binder components.

In the literature, both the presence of carbonaceous residuals and the nature and morphology of these products in the binder-based Mg material produced via sintering are reported to the best of the author’s knowledge. This information is important for understanding the ambiguity observed in cell viability studies, if the additively manufactured Mg materials using binder-based sinter technology are to be qualified for future biomedical applications. This is because the cell viability is dependent on the formed degradation products on the Mg material surfaces, which in turn is dependent on the bulk material microstructure. Therefore, this study aims to prove the presence, distribution and nature of carbonaceous phases formed due to the educts in the final microstructure of MIM Mg-0.6Ca materials.

Detection of carbonaceous residuals remains a challenge for Mg biomaterials processed via the PM route. Due to the possible low-carbon concentrations in the material and high background Mg signals, the energy-dispersive X-ray techniques and X-ray diffraction techniques fail to detect carbon compounds with sufficient accuracy. Commercial devices that detect carbon values in a material using combustion principles cannot be directly adapted for Mg-based materials due to the sublimation properties of Mg upon combustion and possible interference from the formed MgO depending on the test conditions. This indicates the necessity for an in-depth microstructural investigation using advanced material characterization techniques for the sintered MIM Mg-0.6Ca materials and is the scope of the present study to elucidate such carbon impurity phases using a correlative approach.

Scanning electron microscopy (SEM) is known for its suitability for imaging surfaces with high spatial resolution (using secondary electrons (SEs)). On the other hand, by using backscattered electrons (BSEs), it is possible to image and provide information on the material composition from deeper regions of the microstructure. However, specific carbon-related impurities are a challenge to characterize if they are only randomly present on the specimen surfaces, i.e., at random grain boundaries, and not distributed uniformly in the microstructure [28]. In contrast, Raman spectroscopy, an effective and non-destructive spectroscopic method, which has been established over the last 90 years as a standard tool in material chemistry and physics-related scientific fields, is especially sensitive to the characterization of carbon related species. Although it is well known that pure metallic materials are Raman inactive, we expect that Raman spectroscopy can be helpful to disclose compounds, which may be incorporated at, e.g., grain boundaries, during the fabrication process of Mg-0.6Ca specimens because of the non-fully decomposed organic compounds used and their traces as well as possible chemical products of the reactions. Hence, both techniques are highly complementary [29,30]. Therefore, deep correlative spectroscopy and microscopy investigations can provide insights into the composition of the final specimen—to be used as alloys for implant prototypes—and could help explain the possible effects of the impurity elements in the final chemical and structural composition. In this work, we apply a correlative approach between Raman spectroscopy and scanning electron microscopy (SEM) with the aim of identifying carbon-related compounds and their distribution in MIM Mg-0.6Ca materials.

The major goal, in contrast to work published by other groups investigating similar/related materials, is that we focused our attention on the ex situ study to identify regions of the specimen that exhibit Raman signatures related to carbon and carbon compounds [31,32,33]. We expect, in this way, to identify specimen regions on the microscale with potentially cytotoxic products, remnants of our fabrication process. The advantage of the ex situ correlative approach used here would allow us to identify these “critical” regions in a timely manner, already on the “macroscale” with Raman spectroscopy, without increasing the total time for SEM/EDX investigations (if the whole specimen was inspected).

## 2. Materials and Methods

Pure magnesium powder of size fraction <45 µm (Société pour la Fabrication du Magnésium, Martigny, Switzerland) and a master alloy Mg-10Ca powder of size fraction 45–63 µm (Zentrum für Funktionswerkstoffe gemeinnützige GmbH, Clausthal, Germany) are mixed in appropriate weight proportions to form the Mg-0.6Ca alloy powder mix. The chemical analysis of the powders can be found in Table 1. Fe, Cu, Ni and Ca were measured using flame atomic absorption spectroscopy (240 FS AA, Agilent technologies Deutschland GmbH, Waldbronn, Germany), and the remaining elements were measured using Spark atomic emission spectroscopy (SpectroLAB, SPECTRO Analytical Instruments GmbH, Kleve, Germany).

**Figure 1:**
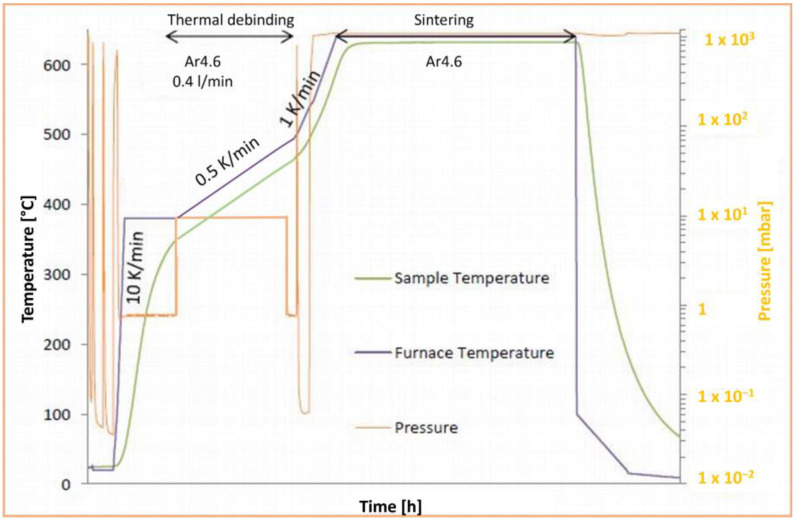
Schematic of the sintering run for MIM Mg-0.6Ca materials used in this study. Thermal debinding and sintering stages are indicated with respect to the pressure changes (orange curves) as function of time.

The process chain to produce Mg-0.6Ca cylindrical specimen in the present work involved the machining of a previously produced Mg-0.6Ca tensile test specimen using MIM technique. Mg-0.6Ca tensile test specimens were subjected to thermal debinding and sintering steps to obtain dense Mg-0.6Ca material, as shown in Figure 1. The Mg-0.6Ca feedstock for MIM technique was produced by adding a 36 vol.% binder system (Table 2) to the Mg-0.6Ca alloy powder mix (64 vol.%) in glass flasks with specially designed Mg lids to avoid contaminations. All the dry components in these flasks were heated to 175 °C for 90 min followed by mixing them thoroughly in a planetary mixer for 5 min at 2000 rpm (Thinky ARE 250 planetary mixer, Tokyo, Japan). The feedstock is allowed to cool down in glass beakers and stored in argon protective glove boxes (MBraun UNILab, Garching, Germany) until further use. For the MIM parts, an industrial injection molding machine (Allrounder 370S, Arburg GmbH + Co KG, Loßburg, Germany) was used to produce tensile test shape specimens according to the standard ISO 2740:2023 [34]. Further details about MIM technique can be found in [35].

The produced green parts were solvent debinded using cyclohexane at 45 °C for 20 h (Lömi EBA50, Lömi GmbH, Großostheim, Germany) to remove the wax and stearic acid components in the binder system. This was followed by a thermal debinding treatment in a hot wall retort furnace (MUT RRO350-900, Jena, Germany) with an increasing temperature from 380 °C to 550 °C with a heating rate of 0.5  K·min^−1^ at a pressure of nearly 5 mbar (Ar 4.6 gas flow of 0.5  L·min^−1^). The sintering followed at a temperature of 644 °C for 16 h under Ar 4.6 at ambient pressure. Details of the sintering setup for PM-processed Mg and its alloys can be found in our other publications [37,38]. A brief schematic of sintering including thermal debinding step is shown in Figure 1.

A cylindrical specimen of diameter 4 mm and height 6 mm was machined out of the head of the sintered tensile test specimen. Cold embedding using a polymeric resin Demotec 30 (Demotec, Nidderau, Germany) facilitated metallographic grinding and polishing. Water-free solutions of alkaline SiO_2_ (0.05 μm particle size in 70% ethanol) and diamond suspension (1 μm particle size in 70% ethanol) were used as agents for polishing on a microfiber cloth. After polishing, the specimens were cleaned with 70% ethanol on a fresh cloth at 50 rpm to remove residual polishing agents sticking to the surface. The same specimen was used for both Raman spectroscopy and SEM analyses, and the experiments were performed consecutively: Raman spectroscopy first and the SEM analyses thereafter. This sequence of characterization methods/this procedure ensures that no unintentional carbon accumulation can occur on the studied surface in the SEM before Raman spectroscopy is carried out. The results with respect to intrinsic carbon contamination would be affected/falsified.

The SEM characterization was performed using the BSE mode (Phenom PRO-X, Phenom-World BV, Eindhoven, The Netherlands) at a source operating voltage of 15 kV at a working distance of 6 mm. Occasionally, low currents and voltages are employed to procure high-resolution images. Energy-dispersive X-ray (EDX) analysis was conducted at map resolution of 512 pixels with 20 ms dwell time. The Raman studies were carried out using a confocal Raman microscope (Renishaw, inVia FSM REFLEX, New Mills, Gloucestershire, UK, Raman spectrometer software-Renishaw WiRE 5) in backscattering geometry. The confocal Raman microscope was equipped with a frequency-doubled Nd-YAG laser (532 nm, 50 mW) and a CCD detector (Renishaw, inVia FSM REFLEX, New Mills, Gloucestershire, UK). The spectrometer was referenced to the transverse optical phonon of Si at 521 cm^−1^. Spectra were recorded in a range between 100 cm^−1^ and 4000 cm^−1^. Great care was taken to overcome any heating effects and/or to prevent unintentional “thermal” damage to the investigated Mg-0.6Ca material/specimen, and, therefore, the laser power was kept below 0.1 mW [39].

## 3. Results

### 3.1. SEM Studies

**Figure 2:**
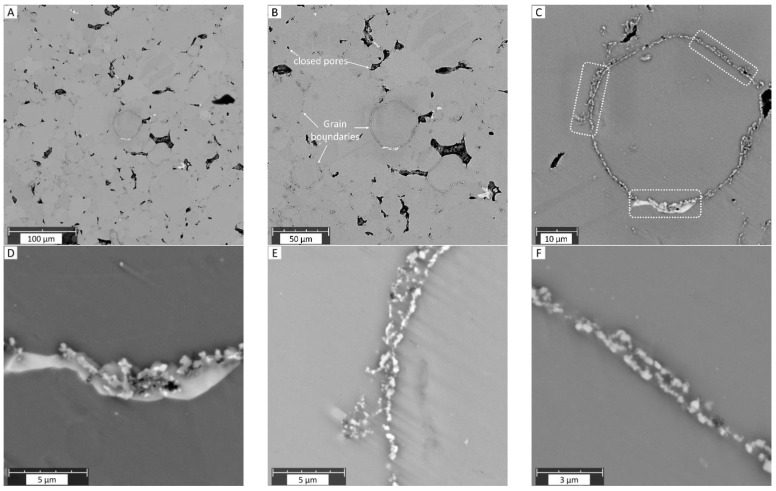
(**A**,**B**) Overview of BSE images of MIM Mg-0.6Ca material. Former particle boundaries and closed porosity can be seen in these images. (**C**) Magnified central area from image (**B**) isolating a single grain in the Mg matrix. (**D**–**F**) Magnified views of selected grain boundary regions indicated with white boxes in image (**C**). The fine comet-like tails in (**C**–**F**) are due to the polishing orientation during metallographic surface preparation.

At a macroscale, the electron microscopy studies revealed a homogeneous microstructure with grain sizes nearing 30 µm and porosity visible at grain junctures (Figure 2). The small pores seemed to have spherical morphologies, while large pores exhibited more complex shapes. The decoration of secondary phases/particles at the grain boundaries is evident (Figure 2B,C). Often, these secondary phases/particles possess sizes <1 µm, visible in Figure 2D–F. No abnormal grain coarsening is observed in the microstructures despite a 16 h long sintering time.

**Figure 3:**
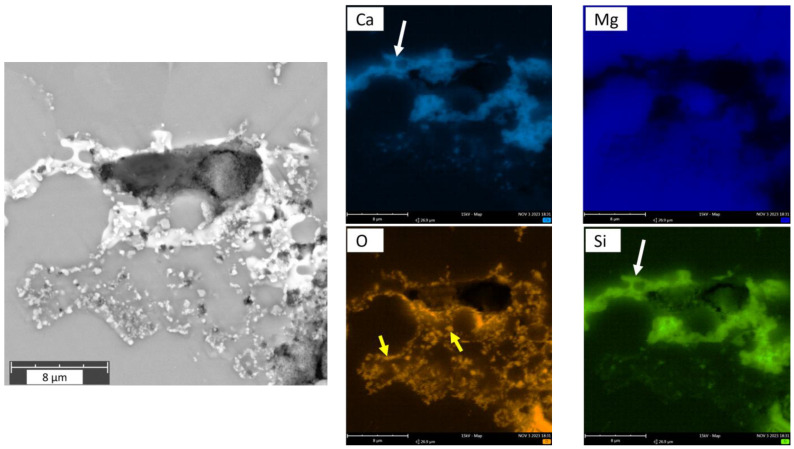
BSE image of MIM Mg-0.6Ca material at a certain grain boundary. EDX elemental maps are shown for Ca, Mg, O and Si for the BSE image. Arrows represent the areas from which elemental signals are emitted with reference to the BSE image.

EDX analysis revealed phases that are rich in Si and Ca, as shown in Figure 3. These phases are randomly formed in the Mg matrix but mainly at the grain boundaries possessing irregular morphologies. Higher magnifications revealed the presence of numerous oxide particles along the grain boundaries. This can be seen in the oxygen element map in Figure 3 and in the magnified images in Figure 2D–F. Most often, it is observed that these oxide-related particles and Ca/Si-rich phases coexist in close vicinity (as shown in Figure 2D and Figure 3 maps) and can only be clearly visualized in the BSE mode. The Ca/Si phase has a slightly higher contrast compared to oxide particles. No carbon-related compounds were discernable from the EDX maps. The presence of O and Si intensities at the bottom-right corner in Figure 3 (pore area) is due to the incorporation of SiO_2_ polishing suspension in certain pores during metallographic preparation, and they are to be considered as an artefact.

### 3.2. Micro-Raman Spectroscopy Studies

**Figure 4:**
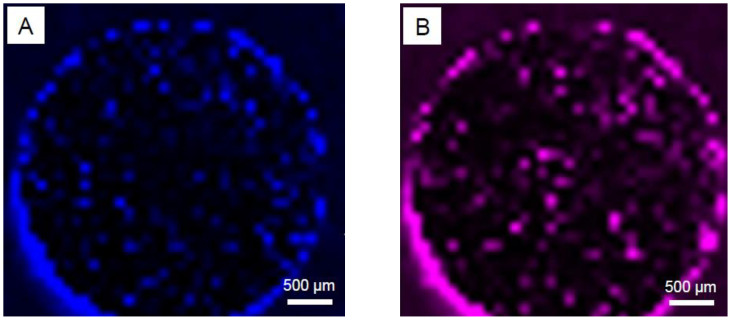
Micro-Raman mapping from the MIM Mg-0.6Ca specimen. Two different carbon-related Raman modes bands (**A**) 1560 cm^−1^ and (**B**) 1865 cm^−1^ are presented here graphically in colors. They are attributed to specific Raman modes of elemental carbon (blue) and to C≡C stretching mode (pink/violet color).

First, we endeavored to obtain an overview of impurities on the polished surface of the whole specimen. To this end, micro-Raman mapping was performed employing an objective lens with 5-fold magnification. Example images of the data collected are presented in Figure 4. Both micro-Raman mappings are based on hundreds of single Raman measurements. The intensity distribution of the maxima for two different Raman modes chosen is shown. Figure 4A represents the intensity at the maximum 1560 cm^−1^ and Figure 4B at 1865 cm^−1^. The intensity is presented here graphically in colors. Due to the surface morphology of the scanned area, i.e., the pores formed during the sintering process as well as the partially strongly reflective (metallic) surface of the specimen, little can be deduced from the absolute intensities recorded at a single/individual specimen position. In addition, due to the objective lens chosen, signals are recorded from a large area. Nevertheless, in all specimen positions, which exhibit high color intensities in both images, both modes are observed, albeit with different intensities/intensity distributions. The collected data at 1560 cm^−1^ and 1865 cm^−1^ for both images presented could be ascribed to different “carbon related compounds.” We assume that they can be attributed to the G-band of elemental carbon in sp2 bonding geometry and to the triple-bonded C≡C stretching mode (for example, in carbides), respectively.

**Figure 5:**
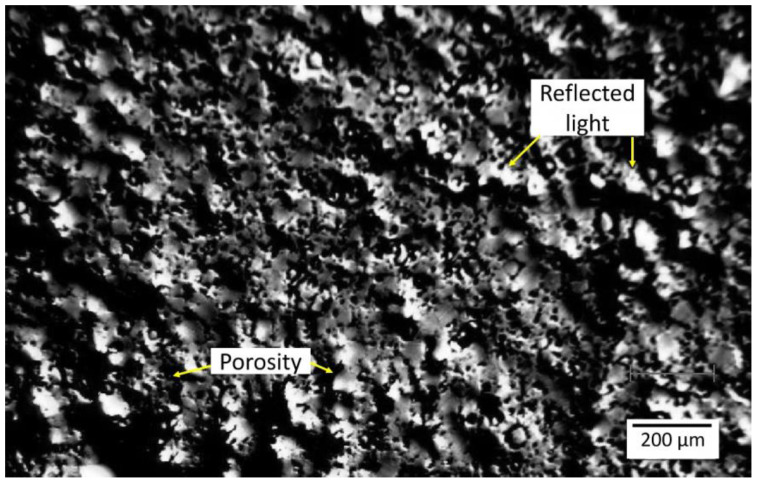
Optical micrograph of the MIM Mg-0.6Ca material from the central position of the specimen on which the corresponding micro-Raman investigations in Figure 4 are presented. The porous surface of the specimen as well as the surfaces of the specimen, which partially reflect light in different directions, are disclosed.

As seen from the optical micrograph in Figure 5, more than half of the total polished surface highly reflects light in different directions. The surface of the specimen appears porous due to the residual porosity that is inherent after sintering treatment of PM materials. Between the highly reflecting light/white areas, dark structures of different morphology are observed.

**Figure 6:**
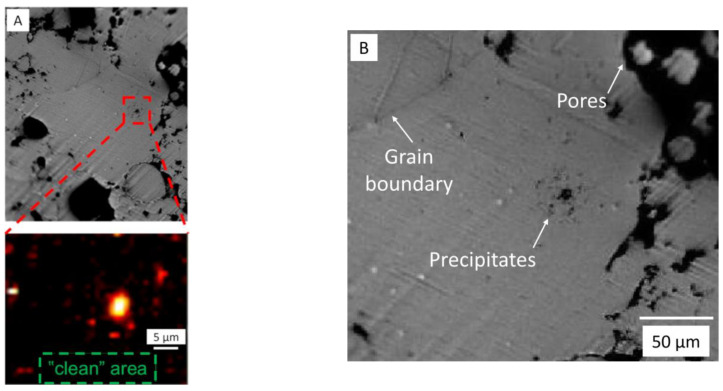
(**A**) Micro-Raman intensity mapping of MIM Mg-0.6Ca material performed at the Raman mode 1560 cm^−1^ with data collected in the target precipitate area indicated in red box. (**B**) Enlarged view of the image (**A**) showing dark precipitates in the grain interiors, grain boundary and pore features.

Consecutively, micro-Raman mapping measurements were performed using a 100-fold objective lens. The aim was to identify which surface morphological features can be correlated with the observed spectra. Figure 6B presents an optical micrograph of the surface area at this magnification, and Figure 6A details the area of the micrograph on which the Raman mapping was performed. In Figure 6A, “clean” (grain interior) and “dark” (precipitate) regions are detected. It is evident that there is a strong contrast between these features in the optical images. The Raman investigations disclose that the recorded spectra of the investigated specimen area are not affected primarily by the different surface morphologies and the reflectivity but rather by the porosity and can be attributed to varying/different chemical compositions in the targeted region. In all cases, the “clean” (grain interior) area seen in Figure 6B exhibits an overall very low Raman signal intensity in the micro-Raman mapping. The “dark” (precipitate) area, in contrast, exhibits distinct signatures with high intensity. Since we are especially interested in carbon impurities, a mapping of the Raman mode at 1560 cm^−1^ typical for carbon-containing compounds is also presented in Figure 6A and confirms that carbon is one important impurity when precipitate areas are investigated.

**Figure 7:**
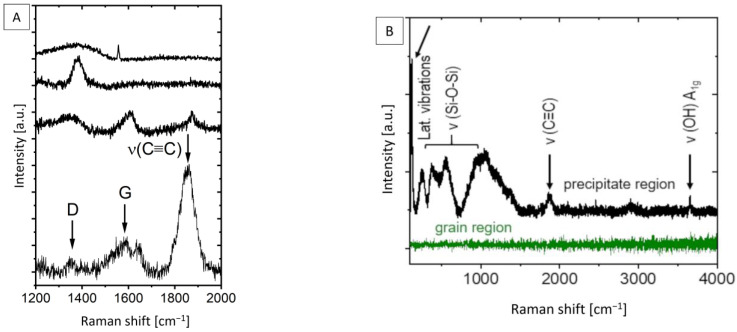
(**A**) Four different representative Raman spectra collected from the “dark” (precipitate) areas on the MIM Mg-0.6Ca material surface in the wavenumber range, in which Raman modes for carbon-related compounds are visible. Typical bands are highlighted with arrows—the D and G bands of elemental carbon as well as the triple-bonded carbon stretching mode. (**B**) Representative Raman spectra for MIM Mg-0.6Ca material collected in the grain interior area (green spectra) and the precipitate area (black spectra).

Four different representative spectra from the “dark” (precipitate) areas shown in Figure 6 as optical images are illustrated in Figure 7A. It centers on the wavenumber range of the spectrum, in which the D and G modes related to amorphous/graphitic carbon are found. It is obvious that the precipitates presented in Figure 6B exhibit, in detail, different signatures indicative of different carbon-related compounds and concentrations. In the spectra, typical important Raman bands are highlighted with arrows—the D and G bands of elemental carbon as well as the additionally observed mode attributed to triple-bonded C≡C stretching vibrations [40]. This additional information can be very useful for the interpretation of correlative data.

Figure 7B presents two typical spectra in the whole wavelength range measured for the grain interior “clean” area (green spectra) and the precipitate “dark” area (black spectra). The “clean” area gave rise to a noisy spectrum without any visible Raman modes. This noisy characteristic of the clean area without visible Raman modes is related to the metallic nature from which the signal stems. Indeed, EDX investigations confirm that Mg is the predominant element detected in the investigated “clean” areas of the specimen (see Figure 3). This result correlates fully with the presented micro-Raman mapping measurements. In contrast, the “dark” (precipitate) areas, which are visible in the central area in Figure 6B, can be attributed to carbon and/or silicon-related compounds. This corresponds to the high-intensity white/yellow/red structure/s in the micro-Raman mapping image in Figure 6A.

## 4. Discussion

The results affirm that Raman spectroscopy is an effective tool for the determination of chemical composition and impurities in materials, in addition to being sensitive to the structural changes and strain evaluations in a large range of materials. Therefore, it is predestined to be one of the core techniques for the correlative characterization of materials together with electron microscopy techniques, as presented in the Results section. However, it must be considered that the materials need to be Raman active, i.e., exhibit photon-induced polarizability of the bonds and/or have structural symmetry without an inversion center. Therefore, the characterization of elemental metals (their alloy composition) cannot be accomplished. In contrast, electron microscopy techniques like SEM in BSE mode are especially suitable for the semiquantitative determination of metal alloy compositions, and, therefore, these two techniques complement each other.

The results presented in our study confirm a direct correlation between the characterization of the microstructure with SEM in BSE mode and Raman spectroscopy studies. Both techniques indicate that “clean” (grain interior) areas of the specimen are Mg metal and are only minimally “contaminated” with carbon-related compounds (Figure 7B green Raman spectra and EDX Map Figure 3). On the other hand, the “dark” (precipitate) areas with different geometrical forms were identified across the whole specimen. The fine spherical precipitates along the grain boundaries may have their origin either from the raw powder stages due to impurities (Table 1) or may have formed during the sintering treatment (Figure 1). The use of a binder system for the MIM technique may allow for additional chemical reactions between the constituents, like Mg, Ca, Si, etc., with the polymeric residue leading to the formation of carbonaceous compounds [41]. Although our characterization techniques were not carried out in the same chamber and at the exact same specimen spot, it was possible to find and identify typical similar objects/morphologies in both characterization techniques and to compare their elemental as well as their chemical composition directly. This is especially the case for the metal hydroxides formed and their characteristic ν (O-H) mode that was identified in micro-Raman mappings (Figure 7B). As Mg is highly sensitive to atmospheric humidity, the observed O-H mode could be related to Mg(OH)_2_ products formed on the surface. Additionally, reactivity with remnant water in the 70% ethanol reagent during the final cleaning step of the polished surface could form this degradation product as well [11,42,43].

Here, in this study, we focused our attention especially on carbon-related compounds and their distribution over the whole specimen area, as presented in Figure 4. We assume that both “elemental carbon” and the “C≡C stretching mode” can be attributed to the products of carbon-containing educts—in our case, the binder system used for specimen preparation (Table 2). Theoretically, the StA, PW57 and PW58 components in the binder system (Table 2) decompose during the solvent debinding step. The solvent debinding efficiency is calculated gravimetrically to be 98–99% in the present work. The rest of the binder system along with the backbone polymer PPcoPE disintegrates at the elevated thermal debinding temperatures prior to sintering. This step should facilitate the removal of decomposed binder fumes from the vicinity of the specimens due to continuous argon filling to the specimen chamber with a simultaneous vacuum suction to maintain medium to low chamber pressure of 5 mbar (Figure 1). During this 7 h thermal debinding stage, however, the decomposed binder fumes might get entrapped in the vicinity of the Mg specimens for a significant amount of time before they are ejected through the vacuum suction. This might facilitate certain high-temperature reactions between the binder fumes and/or backbone polymer with the base material Mg-0.6Ca constituents. Such interactions between metallic bulk and carbon during heat treatment processes affect the final microstructure–property profiles and are also reported for other metallic systems [44]. The literature indicates the formation of metastable MgC_2_ and Mg_2_C_3_-type carbide formations due to reactions between Mg and hydrocarbons. The formation temperatures for MgC_2_ are reported to be between 400 °C and 490 °C and for Mg_2_C_3_ to be between 460 °C and 650 °C, which fall in the thermal debinding temperature range used in the present study [41,45]. However, under vacuum heating conditions, the decomposition of carbides to free carbon is reported to start, for example, for MgC_2_ around 450 °C under vacuum and above 600 °C under ambient conditions, indicating hardly any recovery of carbides at room temperature. The amount of this free carbon formed is additionally reported to be high when PE-type backbone polymer is used in MIM trials, compared to PP-type backbone polymers. The formed free carbon is also reported to have negative effects on the sintering activity due to the formation of a carbon layer on the powder surfaces that prevents interparticle diffusion [46,47,48]. In our study, however, the MIM Mg-0.6Ca feedstock comprises PPcoPE, indicating a mix of PE- and PP-type components (Table 2), and, therefore, the observed Raman mode at 1560 cm^−1^ could be attributed to the remnant free carbon that has been formed because of carbide decomposition at sintering temperatures. The free carbon content formed can, however, be assumed to be low. This is because the density values for MIM Mg-0.6Ca specimens in our study are higher than 95%, indicating high sintering efficiency and, therefore, less hindrance from free carbon during sintering [40].

Due to the reported non recovery of MgC_2_ at room temperature, the C≡C Raman mode cannot be attributed to magnesium carbide. However, studies performed on the structural property revelations of CaC_2_ reported different temperature-dependent polymorphic phases of CaC_2_, like monoclinic and tetragonal, of which the first-order Raman mode at 1860 cm^−1^ was attributed to the tetragonal CaC_2_ phase, and the monoclinic CaC_2_ phase has a reported Raman mode at 1871 cm^−1^ [40,41,49,50]. The Raman band around 1865 cm^−1^ in the present work falls within a window of the spectrum where not many other carbon-related modes for the material under investigation appear. Very often, this region of the spectrum is not even shown. However, in the present work, this mode is distinct, which is typical for a C≡C mode as is in acetylides of Ca. Pressure has been reported to affect the phases, and CaC_2_ polymorphism is often reported leading to a shift in the C≡C stretching bond [40]. In the presented sintering setup, the pore closure occurs during later stages of isothermal sintering. The trapped gas consisting of remnant polymeric fumes inside certain closed pores can lead to local pore pressure variations within the specimens [51,52]. The exact reaction conditions like local pore to matrix pressure, diffusion rate of trapped gas into the neighboring matrix, etc., for the presented sintering conditions are yet unknown; however, they could be the reason for the shifted C≡C stretching mode in the present work. In the literature, the fabrication of CaC_2_, amongst others, was achieved by stoichiometrically mixing the components followed by compaction and solid-state heating to temperatures nearing 1200 °C for 3 h in an induction furnace, and no study concerning CaC_2_ formation under liquid-phase activation conditions nearing 650 °C (like in the present study) was found [40]. At sintering temperatures used in our study, Ca is reported to form CaO due to its low enthalpy of formation (−635 kJ mol-1) compared to Mg (−601 kJ mol-1), leaving oxide-free Mg for sintering [15]. As mentioned previously, carbide dissolution occurs above 600 °C, leaving free C during liquid-phase sintering. Therefore, the feasibility of interactions between CaO and C at the presented sintering conditions of this work requires further systematic investigations.

The different carbon compounds identified (Figure 7) are non-homogeneously distributed, and their specific Raman modes differ significantly (up to several wavenumbers in Raman shift), which is most probably affected by non-uniform local strain distribution in the material [53,54]. These might originate from the dislocations, stacking faults, and pores in the material microstructure [55]. Furthermore, we assume that the different Raman spectra (Figure 7) collected on the precipitate region (Figure 6B) are an indication of the non-homogeneous material production. This could be related to the locally inhomogeneous distribution of the educts such as the binder materials used for the MIM process, leading to different D and G mode intensities, as presented in Figure 7A. Hence, in the future, the fabrication process should be optimized not only for the sake of improvements in the material’s mechanical properties but also with respect to avoiding possible cytotoxic effects. Therefore, the monitoring of the D- and G-carbon-related modes as well as of that of calcium carbide over a large area (Figure 4A,B) seems to be an important issue towards further material optimization.

**Figure 8:**
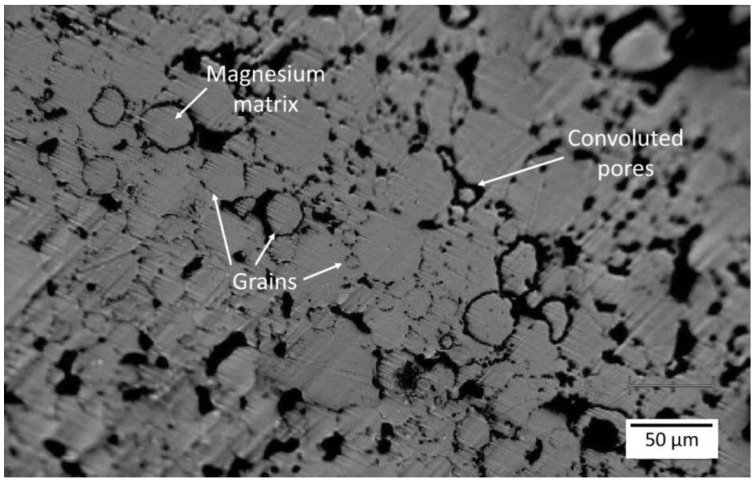
Representative optical microscope image of a larger area of the investigated MIM Mg-0.6Ca material. Features like grains, porosity and former particle boundaries are visible.

On the other hand, grain interiors exhibit a metallic character, and these regions are, therefore, not Raman active. Indeed, analogous/similar characteristics are observed, as presented in Figure 6 (micro-Raman intensity mapping image) and Figure 7 (single micro-Raman measurements) for the metallographic polished “clean” (grain interior) areas. The inhomogeneous size distribution of the spheres is related to the depth at which the grain/sphere was cut during metallographic preparation. The average grain diameter of powder-processed Mg-0.6Ca material was measured to be between 25 µm and 30 µm [56]. It is reported for PM Mg materials that, despite the relatively high sintering temperatures and durations, the grain sizes are restricted because of the native oxide and oxide particles surrounding the starting powders, which, during the sintering process, act as pinning phases at the material grain boundaries. Due to this, the grain sizes are measured to be around 30 µm [19,56]. The grain size distribution for powder-processed Mg-0.6Ca materials can be visualized in [19]. The distribution of the formed secondary phases, on the other hand, is not uniform along every grain boundary at a submicron level. This is especially true for carbon residuals and certain phase segregations along the grain boundaries. This means, despite obtaining a fine grain structure, the actual sintered microstructure is not very homogenous, as shown in Figure 3 and Figure 6 in the form of mapping. Explicitly, the microscope image in Figure 8 shows the grains only partially and grains with smaller diameters appear at different depths corresponding to this cut and metallographic polished plane. Nevertheless, single grains smaller than 10 µm in diameter can be identified in Figure 8. They represent about 20 percent of the total grains over the whole specimen area and are amidst the grains with bigger diameters. The evolution of the coalescence of these grains and simultaneous formation of carbonaceous compounds using different sintering parameters, for example, Argon + 5% H_2_ gas during thermal debinding, will be the subject of our future studies. This could be important because the mechanical and degradation properties of the Mg materials might be strongly affected by different carbonaceous phase formations during sintering.

Si is a common impurity in the form of SiO_2_ in many engineering materials. Also, in the present study, both Mg and Mg-10Ca starting powders contained Si in a concentration of around 200–220 ppm (Table 1). These particles remain inert at the sintering temperatures used in this study and are, therefore, reflected in the sintered microstructures. These are visible as Raman modes at the grain boundaries in Figure 6. Additionally, due to the high oxygen affinity of Mg and Mg-10Ca powders, a metastable MgO layer is reported to form, which is reported to be around 20 nm thick, in addition to impurity oxides like SiO_2_ that form during the gas atomization process [57]. These numerous oxide particles are also inert and are reflected later in the final sintered microstructures, as seen in EDX maps in Figure 3.

## 5. Conclusions

In this work, we performed correlative Raman spectroscopy and SEM investigations on the metal injection-molded Mg-0.6Ca material to evaluate the microstructure and detect impurities, especially carbonaceous residuals due to the processing route. The main findings are as follows:

1. Contrary to the previous reports that PM-processed Mg materials generally exhibit a homogenous microstructure, the current findings show a certain microstructural inhomogeneity in the form of Ca/Si-rich phase and oxide phase segregations, as well as the carbon compound distribution along certain grain boundaries in MIM Mg-0.6Ca material.
2. The sintered MIM Mg-0.6Ca material contains residual carbon products in the form of free carbon and carbides, as confirmed by the stretching modes at ~1370 cm^−1^/~1560 cm^−1^ and ~1865 cm^−1^ from Raman spectroscopy, respectively.
3. These carbon compounds are a result of the reactions between the backbone polymer PPcoPE in the used binder system and the bulk material constituents during the thermal debinding and sintering stages of MIM Mg-0.6Ca material. The thermal decomposition of magnesium carbides at sintering temperatures leads to free carbon in the final material. The detected C≡C stretching mode (~1865 cm^−1^) indicates the formation of CaC_2_.
4. Additionally, the presence of impurities such as SiO_2_ and Ca/Si-rich phases is confirmed by EDX analysis, attributing these phases to the common Si impurity in the starting powders.

This first-of-a-kind correlative working approach is fast, non-destructive in nature, and has potential in elucidating surface or bulk carbonaceous contaminations in Mg biomaterials due to processing conditions. The scientific findings in this work can be employed in the development strategies for new Mg alloys for biomedical applications and link the Mg biocompatibility to the material microstructural features with improved confidence.

## Figures and Tables

**Table 1:** Chemical analysis of the starting pure Mg and Mg-10Ca powders used in this study [19].

Metallic Powders	Elemental Composition [wt.%]							
	Fe	Ni	Cu	Ca	Si	Mn	Zn	Mg
Pure Mg (d_50_ = 22 µm)	0.0018	0.0006	0.0002	0.0041	0.0221	0.0110	0.0027	Bal.
Master alloy Mg-10Ca	0.0021	0.0003	0.0012	10.214	0.0200	0.0150	0.0034	Bal.

**Table 2:** List of binder components used in the present study to produce Mg-0.6Ca feedstock for MIM technique [36].

Binder Component	Abbreviation	Manufacturer
Paraffin wax [50 wt.%]	PW58	Merck
Paraffin wax [10 wt.%]	PW57	Merck
Stearic acid [5 wt.%]	StA	Merck
Polypropylene-copolymer-polyethylene [35 wt.%]	PPcoPE	-- ^1^

^1^ Manufacturer cannot be named due to proprietary interests.

## Data Availability

The raw data supporting the conclusions of this article will be made available by the authors on request due to privacy.
